# A digital transformation and consumption patterns of the “New older adults” in China: a systematic review of technological adaptation and learning mechanisms

**DOI:** 10.3389/fpubh.2025.1717508

**Published:** 2026-03-30

**Authors:** Baowen Shao, Muhammad Inshal, Mirza Amin ul Haq, Syed Taha, Najmonnisa Khan, Jing Wang

**Affiliations:** 1 Shandong Huayu University of Technology, Dezhou, China; 2 Universiti Pendidikan Sultan Idris, Tanjong Malim, Malaysia; 3 Indus Hospital & Health Network, Karachi, Pakistan; 4 Ziauddin University, Karachi, Pakistan; 5 UCSI University, Cheras, Malaysia; 6 University Canada West, Vancouver, BC, Canada; 7 SZABIST University Pakistan, Karachi, Pakistan; 8 School of Culture and Communication, Putian University, Putian, China

**Keywords:** new older adults, aging population, China, technology, market segmentation

## Abstract

China is experiencing a profound demographic shift which is mainly marked by rapid population aging. This systematic review explores the key domains, market segments, and technological innovations targeting China’s new older adults population, employing thematic analysis to synthesize findings from diverse studies. The review identifies multifaceted market segmentation approaches, including demographic, geographic, psychographic, health, and economic channels, which highlights the heterogeneity of older adults consumers. Technological advancements span health and wellness, living environment safety, social inclusion, data infrastructure, and support services, providing benefits such as enhanced health monitoring, independent living, social connectivity, and resource optimization. But current research is constrained by narrow geographic coverage, limited representation of non-digital users, and reliance on theoretical or macro-level analyses, restricting understanding of real-world adoption and long-term outcomes, and future strategies should focus on user-centered technology design, differentiated market segmentation, inclusive digital literacy initiatives, and integrated, AI-enabled service delivery. Also, robust, multi-sector longitudinal studies are needed to evaluate adoption, effectiveness, and scalability of interventions, and this review provides actionable insights for policymakers, industry, and researchers to develop inclusive, efficient, and sustainable support systems for China’s diverse and evolving older adults population.

## 1 Introduction

China is experiencing a profound demographic shift marked by rapid population aging, in 1999, the country officially entered an aging society, in 2021 entered in an aged society, with the proportion of people aged 60 and above reaching 13.5% in 2020 and projected to rise to nearly 30% by 2050, with similar trends in rural areas (1). This demographic transformation is occurring at a much faster pace than in developed countries, i.e., China’s older adults share grew from 10 to 20% in just 25 years, compared to 115 years in France and 85 years in Sweden (2). A distinctive subgroup within this aging population, often termed the “New older adults,” remains relatively active, open to new experiences, and eager to engage socially, with specific demands for personalized and diversified products that meet both practical and emotional needs (3, 4), and this burgeoning demographic represents a powerful new market for industries and policymakers aiming to address their unique needs. As China transitions into a moderately aging society. The older adults population continues to rise significantly, and there is a multifaceted opportunity to explore domains and market segments targeting the New older adults, ranging from healthcare and smart homes to leisure and social participation (5, 61), and this demographic is characterized by active engagement and evolving consumption patterns, creating diverse market demands (3, 4, 61).

With rapid advancements in technology, the role of innovation in serving China’s older adults population has gained prominence. Key technological developments are emerging to address challenges faced by the older adults. These developments include health monitoring devices, eldercare robots, smart home systems, and telemedicine platforms (6, 7), which are not only improving the quality of life for older adults individuals by enhancing safety and independence but are also reshaping the ways industries engage with this demographic (6). In other words, the focus is shifting from basic older adults care to quality senior living via technology and scenario integration, which supports personalized and precise eldercare solutions (6). There are 60,000 types of older adults products available globally, but China accounts for about 2,000 types of older adults products, highlighting a significant gap in supply tailored to the needs of the older adults (8). In terms of benefits, beyond convenience, these innovations indeed contribute to improved health outcomes, reduced caregiver burden, enhanced social connectivity, and greater autonomy for seniors (9). The benefits extend to broader societal gains, such as alleviating pressures on healthcare systems and fostering a more inclusive silver economy (10). Therefore, identifying the core technological domains and understanding their characteristics is crucial for developing comprehensive strategies to meet the evolving needs of the New older adults, along with assessing the benefits of these technological advancements, is essential to appreciating their impact on the older adults population in China.

The purpose of the current systematic literature review is to address existing critical gaps in the current literature by exploring key technological innovations, their key characteristics and benefits, and the market domains they serve through the three key research questions, i.e., What are the key domains and market segments used to target the new older adults market in China? What are the key technological innovations by key domains to serve the new older adults market in China, and what are their core characteristics? What are the key benefits of these technological innovations for China’s new older adults population? Moreover, the research also aims to provide valuable insights by synthesizing the carefully selected studies for businesses, market investors, technology and product developers, elder care institutions, policymakers, and government targeting or serving the Chinese older adults market. The contributions of this paper are as follows: Section 2 of the study deals with the background of the new older adults in China and technological innovations in this context, their key characteristics and benefits. Section 3 of this study deals with research methodology, which includes research questions, search string, screening criteria, study selection process illustrated via PRISMA flowchart, quality assessment (QA) criteria, method for data extraction, keywording-guided screening for full-text analysis, and classification themes. In Section 4, the research results are demonstrated by synthesizing the selected papers and presenting them in the form of tables. In the end, in Sections 5 and 6, a summary of findings and the conclusion of the study are presented.

## 2 Background

The demographic landscape of China has been profoundly shaped by its population policies and rapid economic development. For example, following decades of population control measures such as the one-child policy, which was implemented in 1979 and relaxed in recent years, China faces the unprecedented challenge of a rapidly aging population (11, 63) and resulting many seniors having only one child to rely on, contributing to sharp rise in caregiving dependency ratio (12). The demographic structure is shifting dramatically, with projections indicating the population aged 60 and above will reach approximately 40% by 2050 (13). In 2023, the population aged 60 and above reached nearly 297 million (14), and by 2025, it is estimated to reach more than 300 million (15). This shift creates substantial economic and social pressures, including a shrinking labor force and rising demands for health and social care, constraining economic growth and increasing financial burdens (9, 16).

Simultaneously, this demographic transition has catalyzed the emergence of the “silver economy.” It covers the economic activities and productivity, catering the needs or requirements of older persons and sectors serving aging population (17). The New older adults in China differ markedly from prior generations, exhibiting higher education levels, urban residency, and significant engagement with digital technologies, enabling a more active and demanding consumer group (3, 4), and the concept of the “new older adults” aligns with Neugarten's (18) “young-old” and embodies what Laslett (19) termed the “Third Age,” which is a life stage characterized not by decline but by personal fulfillment, though still shaped by existing social divisions like class and gender (20). This has led to diverse market segments spanning healthcare, smart housing, mobility, leisure, and financial services, triggered by the changing consumption patterns and lifestyle preferences of the older adults (15, 21). Also, the senior economy of China is about 7 trillion yuan (6% of GDP) in 2024, and it is projected to reach 30 trillion yuan (10% of GDP) by 2035 (21). Despite the growing interest, its scope remains fragmented, and not limited to just healthcare, but spanning housing, transport, wellness, and technology (62), yet lacking clear boundaries or systematic segmentation that underscores the need for systematic clarification.

Technological innovation plays a pivotal role in meeting the complex needs of this population. Eldercare robots, wearable health devices, telehealth, and smart home technologies represent key innovations with core characteristics such as enhancing health monitoring, promoting safety and independence, facilitating social connectivity, and alleviating caregiver burdens (22, 23). However, scholarly understanding of these technologies’ deployment, characteristics, and uptake in China’s unique socio-economic context is fragmented across various domains. Moreover, evidence about the tangible benefits these innovations provide to the New older adults, such as, social and health-related economic, remains scattered (7, 24), necessitating a comprehensive synthesis.

Given these significant knowledge gaps, this systematic literature review seeks to identify and clarify the key domains and market segments that target China’s New older adults population firstly, then synthesize evidence on technological innovations within these domains and their defining characteristics, lastly to evaluate the documented benefits arising from these technologies for the older adults population. This inquiry aims to provide clarity and actionable insights to researchers, policymakers, and practitioners engaged in China’s silver economy, supporting more targeted interventions and innovations.

## 3 Research methodology

**Figure 1:**
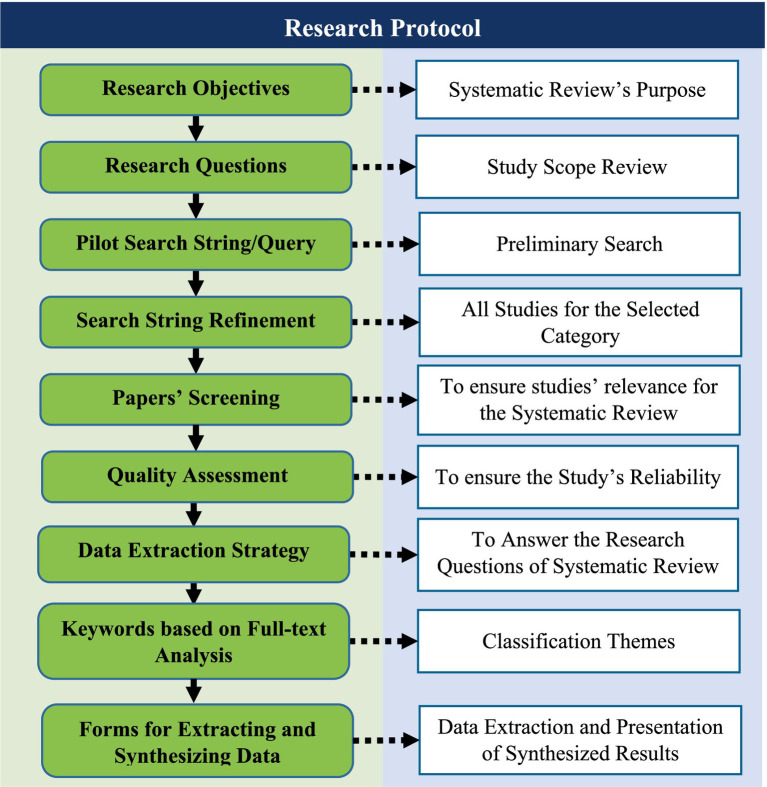
Research protocol.

The current systematic literature review follows a review protocol, and it is illustrated in Figure 1, which outlines the methods for minimizing any potential biases in the study.

The research questions of this systematic literature review, as in Section 3.2, are derived from the study’s objectives in Section 3.1, and the search strategy is formed through pilot searches to obtain a refined search string in Section 3.3, and in order to identify papers in the relevant category. Then, in order to screen the relevant papers, screening criteria are established in Section 3.4. Section 3.5 deals with the study selection process using the PRISMA flowchart, and in Section 3.6, quality assessment (QA) criteria for included studies are discussed. Section 3.7 deals with a data extraction strategy related to collecting data from included studies is formulated. Furthermore, Sections 3.8 and 3.9 deal with keywording-guided screening for full-text analysis and classification schemes. Finally, forms are created to extract data and present synthesized results in the analysis section.

### 3.1 Research objectives

*RO1*: To identify the key domains and market segments used to target the new older adults market strategically in China.

*RO2*: To identify the key technological innovation domains targeting the new older adults market in China and recognize their core characteristics.

*RO3*: To identify the key technological innovation domains targeting the new older adults market in China and their benefits.

### 3.2 Research questions

1. What are the key domains and market segments used to target the new older adults market in China?
2. What are the key technological innovations by key domains to serve the new older adults market in China, and what are their core characteristics?
3. What are the key benefits of these technological innovations for China’s new older adults population?

### 3.3 Search strings

In order to gather relevant studies from a large pool of search results, the selection criteria must meet the objectives of the systematic review. A former study has suggested that a high recall search strategy has the potential to lead to false positives; on the other hand, a precise strategy narrows down the search results (25). At first, in the pilot phase, search strings were created by using Boolean operators, such as “AND” to narrow search results (i.e., all mandatory terms in the search result) and “OR” to broaden results (i.e., in the search result, any term is acceptable). Also, search modifier, such as “intitle:,” was used to improve search sensitivity while sustaining precision. For search query design, the 256-character limits in the search engine of Google Scholar were considered (26). Since it is central for optimal results to design search strings with the right keywords (25). Therefore, to identify appropriate search strings on Google Scholar and Scopus, search strings with the maximum relevant keywords were tested. The final refined search queries obtained are demonstrated in Table 1.

**Table 1:** Search string (technological innovations for the China’s new older adults market).

Source	Search string	Context
Google Scholar	“New older adults” AND (intitle:"China” OR intitle:"chinese”) AND (“Technology” OR “Digital”) AND (“Adoption” OR “Learning”)	Technology for the new older adults generation in China
Scopus	TITLE-ABS-KEY (new older adults) AND TITLE-ABS-KEY (China) OR TITLE-ABS-KEY (Chinese) AND TITLE-ABS-KEY (technology) OR TITLE-ABS-KEY (digital) AND TITLE-ABS-KEY (adopting) OR TITLE-ABS-KEY (learning)	

The Google search in Table 1 was performed on September 1, 2025, and no publication date restrictions were applied to the timeline. Also, Scopus search was performed on November 11, 2025.

### 3.4 Screening of relevant papers

Not all the studies in the search results returned precise relevance to the research questions of this systematic review, therefore, systematic assessment for actual relevance was performed. Accordingly, the search process for the screening of the relevant studies defined by Dybå and Dingsøyr (27) was used, and during the first screening phase, studies were selected based on their titles, and studies unrelated to the research area were omitted, such as articles relevant to China’s new older adults in other contexts, with different meanings than those applied in the technological context, and such papers were reasoned entirely out of scope for the systematic review and were excluded. The second screening phase involves the reading of the abstract of each selected study in the first screening phase, and the inclusion and exclusion criteria were also applied. The following four types of studies were excluded:

1. Studies with no relevance to the search string used.
2. Studies not available in the English language.
3. Studies with non-availability of full text.
4. Studies released outside of conferences, journals, technical reports, patents, and academic theses/dissertations.

Studies, in the end, were selected based on the above exclusion criteria.

### 3.5 Study selection process

**Figure 2:**
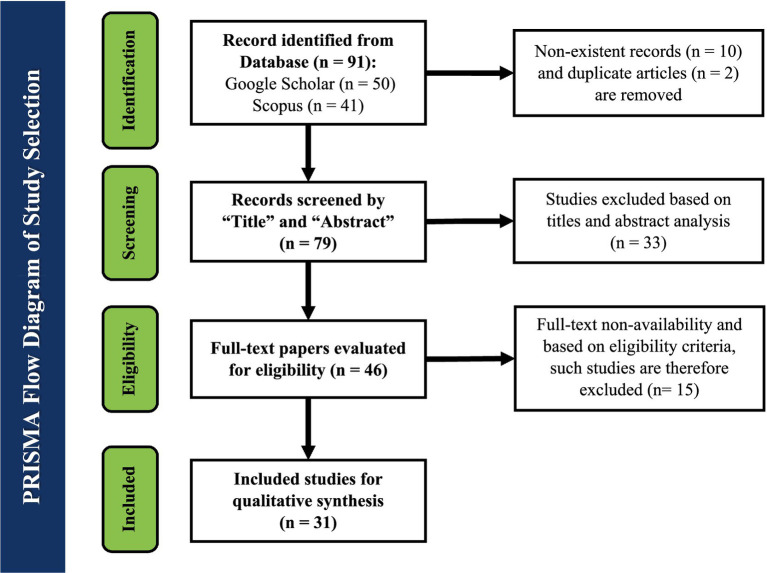
PRISMA flowchart for technological innovations for the China’s new older adults market.

In this systematic review for the selection of eligible studies, focusing on China’s new older adults market in the context of technological innovations, the PRISMA (Preferred Reporting Items for Systematic Reviews and Meta-analyses) flowchart has been used. Initial electronic searches from Google Scholar and Scopus gave a total of 91 records, of which 10 were non-existent records (n = 10) and 2 were duplicated studies. After this exclusion, a set of 79 studies was available for screening, and after screening, 33 studies were further excluded because of irrelevant titles, keywords, and abstracts. From the remaining 46 studies, 15 studies were eliminated further because of full-text unavailability and eligibility criteria. At the end of this process, a set of 31 studies was included in the qualitative synthesis, and the outcomes of the search and selection process are demonstrated in Figure 2 via the PRISMA flowchart.

### 3.6 Quality assessment

A common practice in Systematic Literature Review (SLR) is to conduct quality assessment (QA) in order to appraise the selected papers for their reliability (28). In this SLR, a quality assessment tool was adapted, which was employed in previous mapping studies (28, 29) to evaluate the quality of the selected studies.

The following dimensions were covered by this quality assessment tool:

(a) *Topic Relevance:* Does the selected study discuss the new older adults or other older adults population in China and the characteristics, needs, or preferences of this population group? Yes (+1) or No (0).
(b) *Context-Fit:* Does the study focus on technological innovations for the older adults population in China, including their characteristics or benefits? Yes (+1), Partially (0.5), or No (0).
(c) *Citation Count:* Had the published study been cited by other articles? If the citation count was 1 to 5, it was considered as “Partially (0).” If it had not been cited by any author, it was considered as No (−1). If the citation count was more than five, it was considered as “Yes (+1).”
(d) *JCR Ranking:* What’s the published study’s source? For that, the following Journal Citation Reports (JCR) lists will be taken into consideration while evaluating this (see Table 2).

**Table 2:** Quality criteria.

Sources	Ranking	Score
Journal	Q1	2
	Q2	1.5
	Q3	1
	Q4	1
	Study with no JCR ranking	0

JCR, Journal Citation Reports.

Each selected study for this review is assigned a score for each question of quality assessment tool, and a cumulative score for each study ranges from −1 to 5.

### 3.7 Data extraction method

The potential responses to the defined research questions of this systematic review can be obtained by using the following data extraction strategy:

*RQ1*: The answer to this research question is given by identifying the key domains and market segments used to target the new older adults market strategically in China.

*RQ2*: The answer to this research question is given by identifying the key technological innovation domains targeting the new older adults market in China and their core characteristics.

*RQ3*: The answer to this research question is given by identifying the key technological innovation domains targeting the new older adults market in China and their benefits.

### 3.8 Keywording-guided screening

Petersen et al. (64) used an approach to identify relevant studies by analyzing abstracts with specific keywords. This approach has two stages, i.e., to observe the abstracts first to find the main ideas and keywords that show what the studies were about. In second stage, investigation was done deeper into these keywords to not only understand them better in the context of the research questions but also to organize the reviews in an effective manner by grouping and categorizing the keywords. Further, it was recommended to explore the introduction or conclusion sections in case of a poor-quality abstract (64). In the case of this systematic review, the full text was analyzed for robust classification themes.

### 3.9 Classification themes

The methodology was adapted from Petersen et al. (64) for the classification themes of the domain-specific segmentation of China’s new older adults market, technological innovations targeting this market, their key characteristics and benefits as presented below in Tables 3–5.

**Table 3:** Classification themes: segmentation of China’s new older adults market.

Research question	Classification	Themes	Sub-themes	Sub-sub-themes
RQ1	“New older adults” market segmentation	Demographic segmentation	Age and life stage	Individuals born in the 1960s
				Middle-aged adults
				Older adults
				Seniors (50/55+)
				Older adults (with limitations)
			Occupation	State agency heads
				Farmers/workers
				Enterprise employees
				Private owners
				Freelancers
			Income level	Income level and purchasing power
				Low-income
				Lower-middle-income
				Middle-income
				Higher-middle-income
				High-income
			Population density	Targeting high-density older adults populations
			Education level	Senior universities
				Different education level
			Socioeconomic status	Lower economic advantage
				Lower education attainment
				Special needs groups (disabled and poor older adults, older adults living alone)
		Geographic segmentation	Urban vs. rural	Development level
				Resource access & needs
				Rural middle-aged/older adults vs. urban older adults
				Targeting to bridge the urban–rural consumption gap
			Regional development	Economic development level
				Less-developed vs. commercial cores
				Remote areas, ethnic areas, backward areas
				Central urban districts vs. newly developed urban areas
				Urban core vs. township areas
			Location type	Urban residents vs. Rural residents
				Urban centers vs. Remote suburbs
				County-level areas
				Urban–rural integration zones
			Accessibility	Seniors within a 5 to 15-min walkable life circles
				Areas with medical service gaps
		Psychographic segmentation	Attitudes and motivation	Strong desire for self-improvement and social interaction
				High intrinsic motivation
				Forward-looking and self-developing
				Individuals seeking independence
				Individuals seeking reduced dependency
				Constructive user of leisure time, entertainment, and social connection
				Individuals seeking to improve proficiency
				Preference for comfort and safety
			Lifestyle and values	Prioritizing travel and connectedness with family/friends
				Value comfort, convenience and safety
				Seeking a diversified life choice
				Individuals interested in lifelong learning
				Supports “active aging”
				Self-realization
				Dignity
			Mindset	Individuals seeking escapism
				Individuals seeking self-cognition
			Outlook	Attitude toward new things
		Behavioral segmentation	Product usage	Preference for “Older adults Mode” apps
				Preference for family version apps
				Traditional booking methods (travel agencies/ telephone)
			Technology adoption and usage	Digital literacy
				Digital self-efficacy
				Struggling to keep pace
				Smartphone ownership
			Travel and consumption	Preference for off-season travel
				Price sensitivity and value-seeking behavior
				Travel with family/neighbors/friends.
			Usage preferences	Preference for investments and social networking
			Engagement	Users seeking mental health knowledge
				Users of online courses
				Users of cognitive tools
				Users of digital cultural products (online chess, drama, cloud social networking)
				Users of digital platforms for transactions and financing
			Engagement frequency	Learning frequency
				Learning motivation
			Purchasing behavior	Budget-consciousness
		Health and dependency segmentation	Health status	Disabled older adults (moderate, mild, severe)
				Disabled and Semi-disabled older adults
				Dementia older adults
				Older adults with chronic diseases
				Older adults with “Sub-healthy” conditions
				Older adults with disabilities cognitive/physical
				Individuals needing rehabilitative benefits
			Cognitive/physical ability	Decline in learning skills, visual acuity, memory
				Mobility limitations
				Need for medical support and barrier-free facilities
			Care needs	Rehabilitation care
				Hospice care
				Post-illness rehabilitation
				Daily nursing care
			Dependency level	Individuals requiring long-term care
				Need for integrated medical & pension services
		Service and product benefit segmentation	Service type	Smart eldercare
				Tourism eldercare
				Medical care integration
				Daily care
				Spiritual comfort
				Parks & fitness centers
				TCM services
				Home-based care
				Emergency, standard and scheduled services
				Older adults facilities
				Community dining
				Corporate catering
			Technology integration	Need for IoT, AI, big data and cloud computing
				Remote monitoring
				Telemedicine
				Wearable devices
			Experience sought	Immersive experiences
				Leisure and entertainment
				Learning opportunities
				Cultural activities
			Functional need	Health products
				Intelligent health management
				Mental health knowledge
				Rehabilitative benefits
				Intelligent life care
				Interface simplicity
				simplified processes
				Applying XR to improve quality of life
				Historical and cultural tourism
				Natural landscape tourism
				Spiritual, cultural, and material life needs
			Care model	Home-based care
				Institutional care
				Community and ecological care
		Economic and channel segmentation	Funding and payment method	Long-term care insurance
				Self-pay
				Commercial insurance
				Membership-based model
				Government-run vs. social-run
			Collaborative models	Partnerships for point accumulation with banks, insurers and merchants
			Living arrangement	Home-based
				Community-based
				Institutional
			Economic capacity	Cost-conscious solutions for “working families”
				High-end, vacation-style retirement models.
			Channel preference	Digital channels for transactions, financing, and social connection
				Traditional channels like travel agencies, telephone, in-store bookings
				Family-linked channels for payment assistance
			Enterprise scale	Small business
				Medium-sized enterprise
				Large enterprises; Leading enterprises

**Table 4:** Classification theme: technological innovations targeting the new older adults market in China and their core characteristics.

Research question	Classification	Themes	Sub-themes	Sub-sub-themes
RQ2	Characteristics of technological innovations for China’s older adults market	Health and wellness technologies	Smart health monitoring and wearable devices	Real-time health monitoring
				User-friendly design
				Continuous data collection
				Integration with platforms and third-party devices
			Intelligent health monitoring & management systems	Smart wearable devices
				Multi-sensor data platforms (ECG, pulse oximeter, EMG)
				Remote homecare systems
			AI-powered safety and behavior recognition systems	Deep learning-based fall detection (CNNs, transfer learning)
				Behavior recognition technology (using RGB cameras)
			Telemedicine and remote consultation systems	Video consultations
				Rapid telephone consultation
				Chronic disease management
				Internet medical resources
			Intelligent delivery and nutrition management systems	Smart meal systems
				Online ordering
				Nutrition assessment
				Delivery dispatch
				Multimodal kitchen-terminal tech
		Living environment and safety technologies	Smart home and IoT-based care systems	Sensors
				Monitoring devices
				Voice recognition
				Motion capture
				Integrates hardware and software
			AI-driven predictive safety and resource allocation	Predictive modeling
				Spatio-temporal data fusion
				Dynamic thresholding
				Graded early warning
			Assistive robotics for daily living	Physical assistance robots
				Social robots
				Feeding Assistive Robotics (using HCI)
		Social inclusion and empowerment technologies	Mobile and digital learning platforms	Systematic learning content
				Easy-to-use interface
				Intuitive UI
				Voice navigation
				Features for emotional assurance
			Digital finance and e-commerce integration	Mobile payment
				Low cost
				Credibility
				Digital virtual currency/points
				Integration with e-commerce and real estate
				Digital platforms & smart logistics
				Mobile payment systems (Alipay, WeChat Pay)
				Payment security features & family account integration
			Social connection and communication platforms	Social communication platforms
				Use of cell phones, tablets, and communication software
			Online education and lifelong learning platforms	E-learning platforms for older adults
				Platforms for skills and education
				Digital devices for learning (TVs, mobile phones)
				New Media & Multimedia Applications
			Extended reality (XR) and immersive technologies	Gesture Interaction
				Optimized Gesture Set for the older adults
				Immersive Learning Environments
			Video communication and social connection systems	Audio-video monitoring
				Mobile app integration
				Simplified UI
		Data infrastructure and platform management	Centralized data and information management platforms	Cloud-based centralized databases
				Health data platform
				Four-level linkage mechanism (city, district, street, community)
				Role-based access control
			Service matching and digital marketplaces	Digital platforms
				Online booking
				Feedback mechanisms
			GIS and data-driven spatial planning	High-resolution spatial data (POI)
				GIS integration
				Predictive site selection
			Data-driven analytics and smart service integration	Geospatial modeling and semantic segmentation
				AI algorithms for tourism (machine learning, NLP)
				Constrained Clustering Algorithms
				Care Greeting Network & Matching Model
				Integrated “micro-clinic + smart pharmacy” networks
			User-centered and aging-adapted interaction design	User-Centered Design
				Intuitive Human-Computer Interaction
				High cognitive compatibility & low physiological load
				Adapted product presentation (fonts, colors)
		Support and operational technologies	Digital promotion and self-media platforms	Use of graphics and short videos for promotion
			Privacy-preserving computing	Federated learning
			Foundational digital access infrastructure	Basic connectivity networks
				Age-suitable terminal products
				Simplified software interfaces (“Older adults Mode”)
				Guided tutorials

**Table 5:** Classification theme: technological innovations targeting the “New older adults” market in China and their benefits.

Research question	Classification	Themes	Sub-themes	Sub-sub-themes
RQ3	Key benefits of technological innovations for China’s older adults market	Health and wellness technologies	Smart health monitoring and wearable devices	Improved safety
				Independent living
				Reduced hospitalization
				Real-time alerts
				Health tracking
			Intelligent health monitoring and management systems	Dynamic health information
				Improved quality of life, physical, and mental health.
				Immediate assistance (e.g., fall detection).
				Comprehensive health monitoring.
				Allows ageing in place
				Enables professional care at home.
				Reduces hospitalizations and readmission rates through early detection.
				Enables self-monitoring and management of chronic diseases
			AI-powered safety and behavior recognition systems	Timely detection of abnormal situations (e.g., falls).
				Enhanced safety and security in everyday life.
				Aims to ensure the safety of the older adults living alone.
				High accuracy and speed in recognition.
				Improved service quality in older adults care.
			Telemedicine and remote consultation Systems	Accessible healthcare
				Remote diagnosis
				Accommodates rural service imbalances
				Reduces travel and cost
			Intelligent delivery and nutrition management systems	Enhanced convenience
				Better dietary matching
				Improved nutrition
				Supports sustainable operation
		Living environment and safety technologies	Smart home and IoT-based care systems	Real-time monitoring
				Risk prevention
				Improved service quality
				Remote supervision
				Enhances independent living
			AI-driven predictive safety and resource allocation	Early warning of resource shortages
				Optimized resource allocation
				Supports operational planning
			Assistive robotics for daily living	Assist with activities of daily living
				Improve gait function
				Promote independence and convenience
				Maintain social connections
				Improve spiritual life
				Promotes independence
		Social inclusion & empowerment technologies	Mobile and digital learning platforms	Continuous self-education
				Integration into society
				Social inclusion
				Sense of accomplishment
			Digital finance and e-commerce integration	Accessible & efficient financial services
				Easier saving & investing, profitable options
				Brings convenience
				Reduces depression by narrowing the urban–rural living standards gap
				Provides convenience
				Reduced property security risk
				Enabling online shopping despite a lack of digital payment confidence
			Social connection & communication platforms	Provides “remote companionship”
				Reduces loneliness
				Maintains social connections
				Improves cognitive ability
				Offers access to mental health knowledge
			Online education & lifelong learning platforms	Provided access to high-quality educational resources
				Maintained lifelong learning from home
				Increases income
				Enhances sense of career accomplishment
				Enabled flexible
				Enabled self-arranged learning time and content
				Creates new opportunities for the sustainable development of older adults education
				Helps the older adults learn new knowledge, master new technologies, and integrate into society
				Provided access to high-quality educational resources
			Extended reality (XR) & immersive technologies	Intuitive and low learning curve
				Enhanced quality of life
				Promotes independence and social connection
				Reduced learning difficulty
				Reduced risk of musculoskeletal disorders
				Helps deepen the learning and application of digital cultural products
			Video communication & social connection systems	Remote family connection
				Emergency detection
				Mental support
				Easy access to communication
		Data infrastructure & platform management	Centralized data & information management platforms	Better care coordination
				Accessible anywhere
				Improves resource matching
				Enables integrated service delivery
			Service matching & digital marketplaces	Reduces transaction costs
				Improves purchasing ability
				Facilitates comparison & selection of services
			GIS & data-driven spatial planning	Objective site selection for care facilities
				Improved spatial equity
				Enhanced accessibility
			Data-driven analytics & smart service integration	Analyzes spatial characteristics
				Predicts optimal sites for older adults care facilities
				Assesses walkability
				Precise marketing and personalized travel packages
				Improved efficiency & quality for industrial chain optimization
				Optimal resource allocation to efficiently connect caregivers with the older adults
				Aims to enhance basic healthcare provision in underserved rural areas
			User-centered & aging-adapted interaction design	Accommodates the older adults’s physical and psychological state
				Natural, easy-to-use, and humanized product interfaces
				Makes the technology easier to adopt
				Minimizes physical strain from repetitive use
				Makes products more suitable for the older adults
		Support & operational technologies	Digital promotion & self-media platforms	Increases public awareness
				Promotes culture & services
				Boosts engagement
			Privacy-preserving computing	Protects data privacy
			Foundational digital access infrastructure	Provides the foundational access that enables all other digital services
				Reduces the “digital divide”
				Improved convenience for the older adults to enter the online world
				Enhanced usability and reduced complexity
				Improved learnability and confidence

## 4 Analysis

In this analysis section, the results associated with the research questions of this review are discussed, and after careful selection of the research studies via the screening process, the responses to each research question have been utilized to provide answers. This section has four main sub-sections: the first sub-section deals with RQ1, which explores the domain-specific segmentation of China’s new older adults market, the second sub-section addresses RQ2 and deals with technological innovations targeting this market, their key characteristics, and the third sub-section addresses RQ3 and deals with the benefits of technological innovations. In the end, the fifth sub-section is about the overall quality assessment score.

### 4.1 Key domains and market segments targeting China’s new older adults market

The adapted method for the classification themes (64) is applied to 31 extracted articles through the PRISMA method for the identification of key domains and market segments targeting China’s new older adults market, as indicated in Table 6.

**Table 6:** Classification: domain-specific segmentation of China’s new older adults market.

Domain-specific segmentation of China’s new older adults market								
RQ1								
References	Domain	Demographic segmentation	Geographic segmentation	Psychographic segmentation	Behavioral segmentation	Health & dependency segmentation	Service & product benefit segmentation	Economic & channel segmentation
Shao et al. (30)	Older adults Education Technology (EdTech)	*Age & Generation:* Individuals born in the 1960s *Education Level:* Different Education Level		*Attitudes & Motivation:* Strong desire for self-improvement & social interaction; High intrinsic motivation; Forward-looking and self-developing *Outlook:* Attitude toward new things	*Technology Adoption & Usage:* Digital literacy; Digital self-efficacy *Engagement Frequency:* Learning motivation; Learning frequency			
He et al. (36)	Older adults care services (ECS)		*Urban vs. Rural:* Resource access & needs				*Service Type:* Smart eldercare; Tourism eldercare; Medical care integration	*Funding & Payment Method:* Government-run vs. social-run
Shao et al. (51)	Older adults care resource allocation	*Education Level:* Senior Universities					*Experience Sought:* Cultural activities; Learning opportunities	
Yan (37)	Home older adults care industry					*Care Needs:* Post-illness rehabilitation; Daily nursing care *Dependency Level:* Need for integrated medical & pension services	*Service Type:* Home-based care *Technology Integration*	*Funding & Payment Method:* Government-run vs. social-run; Membership-based model *Living Arrangement:* Home-based; Institutional
Zhou and Ye (38)	Digital Inclusive Finance (FinTech)		*Regional Development:* Less-Developed vs. Commercial Cores		*Technology Adoption & Usage:* Struggling to keep pace; Smartphone ownership *Usage Preferences:* Preference for investments & social networking			
Wang et al. (59)	Urban Planning						*Service Type:* Parks & fitness centers	
Sun (41)	Property-based older adults Care	*Population Density:* Targeting high-density older adults populations	*Location Type:* Urban–rural integration zones; County-level areas; Urban centers vs. remote suburbs				*Service Type:* Emergency, standard & scheduled services	
Yingjie and Huayao (42)	Senior Tourism Industry			*Lifestyle & Values:* Prioritizing travel & connectedness with family/friends; Value comfort, convenience & safety; Seeking a diversified life choice	*Purchasing Behavior:* Budget-consciousness		*Service Type:* TCM services *Experience Sought:* Immersive experiences; Leisure & entertainment	
Yichen (46)	Smart Home Care (SHC) Technology					*Care Needs:* Daily nursing care; Rehabilitation care	*Service Type:* Daily care; Rehabilitation; Spiritual comfort *Technology Integration:* Need for IoT, AI, big data & cloud computing; Remote monitoring; Telemedicine; Wearables	*Funding & Payment Method:* Long-term care insurance; Self-pay; Commercial insurance *Living Arrangement:* Home-based; Community-based; Institutional
Akmal et al. (31)	Older adults Welfare Cottage Industry	*Income Level:* Lower income	*Urban vs. Rural:* Development level; Resource access & needs *Location Type:* Urban vs. Rural residents					
Jiang (45)	Integrated Medical and Care Service System					*Health Status:* Disabled & Semi-disabled older adults; Dementia older adults; Older adults with chronic diseases *Care Needs:* Rehabilitation care; Hospice care	*Service Type:* Home-based care	
Tan et al. (32)	Social Service Incentive Mechanisms	*Income Level:* Low-income, lower-middle-income, middle-income, higher-middle-income, high-income	*Location Type:* Urban residents vs. Rural residents; Urban vs. Rural residents					*Collaborative Models:* Partnerships for point accumulation with banks, insurers & merchants
Zheng et al. (39)	Meal Assistance Service	*Population Density:* Targeting high-density older adults populations	*Regional Development:* Economic Development Level; Less-Developed vs. Commercial Cores *Location Type:* Urban centers vs. Remote suburbs				*Service Type:* Older adults facilities; Community dining; Corporate catering	
Wan et al. (34)	Agriculture & Digital	*Age & Life Stage:* Middle-aged adults; Older adults *Socioeconomic Status:* Rural middle-aged and older adults	*Urban vs. Rural:* Rural middle-aged/older adults vs. urban older adults; targeting to bridge the urban–rural consumption gap	*Attitudes & Motivation:* Individuals seeking to improve proficiency	*Engagement:* Users seeking mental health knowledge; Users of online courses; Users of cognitive tools; Users of digital platforms for transactions and financing		*Functional Need:* Mental health knowledge	*Channel Preference:* Digital Channels for transactions, financing, and social connection
Chen and Hu (50)	XR Technology						*Functional Need:* Applying XR to improve quality of life	
Shen et al. (47)	Healthcare Devices					*Health Status:* Older adults with chronic diseases; Disabled older adults (moderate, mild, severe)	*Functional Need:* Intelligent health management; Intelligent life care	
Gao et al. (40)	Cultural Technology	*Income Level:* income level and purchasing power *Socioeconomic Status:* Special needs groups (disabled and poor older adults, older adults living alone)	*Regional Development:* Remote areas, ethnic areas, backward areas		*Engagement:* Users of online courses; Users of cognitive tools; Users of digital cultural products (online chess, drama, cloud social networking)			
*Zhang et al.* (55)	*Care Monitoring*					*Health Status:* Older adults with chronic diseases *Disability & Dependency Level:* Individuals requiring long-term care	*Care Model:* Home-based Care; Institutional Care *Functional Need*: Health products	
Lam and Lee (35)	Social Services	*Age & Life Stage:* Seniors (50/55+) *Socioeconomic Status:* Lower economic advantage; Lower education attainment		*Lifestyle & Values:* Individuals interested in lifelong learning *Attitudes & Motivation:* Individuals seeking independence; Individuals seeking reduced dependency; Constructive user of leisure time, entertainment, and social connection		*Health Status:* Individuals needing rehabilitative benefits	*Functional Need:* Rehabilitative benefits	
Cheng et al. (58)	Education							
Zhang (33)	Tourism	*Age & Life Stage*: Seniors (50/55+), Older adults (with limitations) *Occupation*: State agency heads, farmers/workers, enterprise employees, private owners, freelancers *Income Level:* Income level and purchasing power		*Attitudes & Motivation*: Preference for comfort and safety	*Product Usage*: Preference for traditional booking methods *Travel & Consumption*: Preference for off-season travel; Price sensitivity and value-seeking behavior; Travel with family/ neighbors/ friends.	*Cognitive/Physical Ability*: Mobility limitations; Need for medical support and barrier-free facilities	*Functional Need*: Historical and cultural tourism; Natural landscape tourism	*Channel Preference*: Traditional Channels like travel agencies, telephone, in-store bookings
Yu and Dong (48)	Urban Planning		*Urban vs. Rural:* Urban core vs. township areas; central urban districts vs. newly developed urban areas. *Accessibility*: Seniors within a 5- to 15-min walkable life circles; Areas with medical service gaps			Health Status: *Older adults with chronic diseases*	Functional Need: *Health products*	Economic Capacity: *High-end, vacation-style retirement models*
Cheng (57)	Education							
An et al. (56)	Older adults-Care Services							
Huang et al. (53)	Assistive Robotics							
Hu et al. (49)	Digital Healthcare					*Health Status:* Older adults with chronic diseases; Older adults with “Sub-healthy” conditions; Disabled older adults (moderate, mild, severe)	*Care Model:* Home-based Care; Institutional Care	
Zhang (43)	Education			*Lifestyle & Values:* Supports “active aging”; self-realization; Dignity			*Functional Need:* Spiritual, cultural, and material life needs	
Zhao and Gai (60)	Older adults Care Services						*Care Model:* Home-based Care; Institutional Care; Community & Ecological Care	*Enterprise Scale*: Small business; Medium-sized enterprise; Large enterprises; Leading enterprises
Wang (44)	New Media			*Mindset:* Individuals seeking escapism; Individuals seeking self-cognition	*Product Usage:* Preference for “older adults Mode” apps; Preference for family version apps		*Functional Need:* Interface simplicity; simplified processes	*Channel Preference:* Family-Linked Channels for payment assistance.
Yun (52)	Health & Wellness Technology	*Socioeconomic Status:* Special needs groups (older adults living alone)				*Health Status:* Older adults with chronic diseases; Older adults with disabilities *Cognitive/Physical Ability:* Decline in learning skills, visual acuity, memory		
Chen and Chen (54)	Care Technology						*Care Model:* Home-based Care *Functional Need:* Health products; Intelligent health management; Intelligent life care	*Economic Capacity:* Cost-Conscious Solutions for “working families”

#### 4.1.1 Assessment of RQ1: what are the key domains and market segments used to target the “new older adults” market in China?

**Figure 3:**
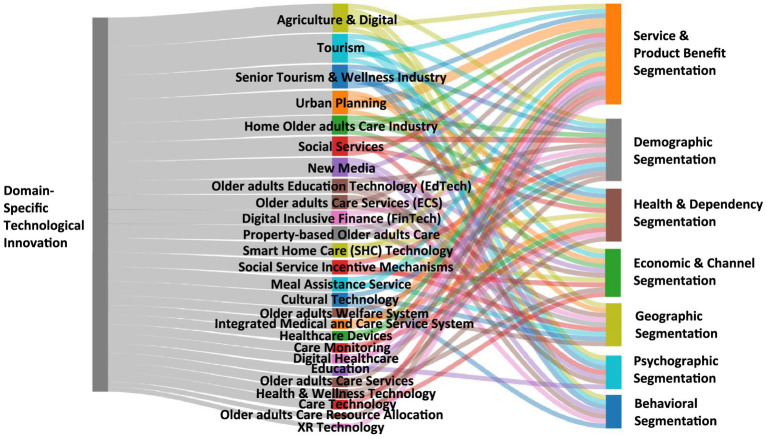
Correlation of technology innovations & market segments targeting China’s new older adults.

The reviewed literature in Table 6 collectively applies a framework to understand the diverse needs or characteristics of China’s new older adults population across various domains, as shown in Figure 3.

1. *Demographic Segmentation.* Demographic factors are widely used across studies, particularly age, education level, income level, occupation, and socioeconomic status. Shao et al. (30) classify learners in EdTech based on generational cohorts (1960s-born) and education levels. Studies such as Akmal et al. (31), Tan et al. (32), and Zhang (33) incorporate income and purchasing power to differentiate market opportunities in welfare services, social incentives, and tourism. Other studies, including Wan et al. (34) and Lam and Lee (35), segment populations based on life stage, highlighting middle-aged adults transitioning into older adulthood.
2. *Geographic Segmentation.* Geographic segmentation plays a central role, especially in older adults care and planning. Several studies differentiate users based on urban vs. rural residency (31, 32, 36, 37), recognizing disparities in resource access and consumption behavior. Others, such as Zhou and Ye (38), Zheng et al. (39), and Gao et al. (40), segment according to regional economic development, targeting less-developed, remote, or ethnic areas. Sun (41) and Yingjie and Huayao (42) introduce finer segmentation such as urban–rural integration zones, county-level areas, and high-density older adults clusters, especially relevant to property-based care and tourism.
3. *Psychographic Segmentation.* Psychographic factors highlight evolving values, motivations, and lifestyles among the new older adults. Shao et al. (30) emphasize self-improvement, openness to new experiences, and intrinsic motivation among EdTech learners. Yingjie and Huayao (42) capture the increasing desire for comfort, safety, family connectedness, and diversified lifestyles in senior tourism. Lam and Lee (35) and Zhang (43) identify psychographic traits related to lifelong learning, independence, active aging, and self-realization, showing a shift away from passive retirement.
4. *Behavioral Segmentation.* Behavioral segmentation is prominent across digital, financial, and tourism domains. Shao et al. (30) and Zhou and Ye (38) examine technology adoption, digital literacy, smartphone ownership, and engagement patterns, which influence participation in EdTech and FinTech. Yingjie and Huayao (42) highlight budget-conscious purchasing behavior among older adults tourists. Wang (44) segments users based on media consumption behaviors, such as preferences for “Older adults Mode” apps or family-linked features for payment support.
5. *Health & Dependency Segmentation.* Health status, chronic disease presence, disability levels, and dependency needs are critical segmentation criteria. Yan (37), Jiang (45), Yichen (46), and Shen et al. (47) emphasize chronic disease management, post-illness rehabilitation, dementia care, and varying disability levels, which correspond to differentiated service models (home-based, institutional, hospice). Studies such as Yu and Dong (48) and Hu et al. (49) further categorize older adults users by sub-healthy conditions and mobility limitations, which heavily influence service design and accessibility requirements.
6. *Service & Product Benefit Segmentation.* Across sectors, segmentation involves the benefits or experiences older adults users seek. He et al. (36) and Yan (37) distinguish between smart eldercare, tourism eldercare, and integrated medical care. Yichen (46) and Chen and Hu (50) identify demand for IoT-enabled home care, telemedicine, rehabilitation, spiritual comfort, and XR-based solutions. Studies in culture and technology (40, 51) segment users by desired experiences such as cultural learning, online entertainment, cognitive tools, and immersive activities. Property-based care (41) and tourism (33) highlight expectations regarding emergency services, safety, historical or cultural experiences, and leisure amenities.
7. *Economic & Channel Segmentation.* Economic segmentation appears through analysis of income, funding mechanisms, insurance coverage, and enterprise scale. He et al. (36) and Yan (37) differentiate between government-run vs. social-run models. Tan et al. (32) incorporate income tiers into social incentive mechanisms involving banks and insurers. Yichen (46) details multiple payment channels including self-pay, long-term care insurance, and commercial plans. Channel segmentation also emerges in studies like Wang (44) and Zhang (33), which highlight traditional vs. digital channels, including family-linked payment support, travel agencies, and in-store bookings.

To conclude, the reviewed studies collectively demonstrate that China’s new older adults market is deeply heterogeneous. As it is characterized by diverse demographic attributes, like socioeconomic backgrounds, geographic contexts, lifestyle values, digital behaviors, health conditions, and service expectations, and the segmentation patterns reflect a shift toward viewing the older adults not as a uniform group but as multidimensional consumers with evolving needs that cut across technology, healthcare, tourism, education, welfare, digital finance, and property-based services. This multidimensional segmentation highlights the need for tailored, domain-specific strategies in policy, product development, and service delivery, instead of “one-size-fits-all” approaches. The effective interventions for China’s new older adults must account for generational differences, urban–rural disparities, digital readiness, health variations, lifestyle aspirations, and economic capacity, ensuring inclusive and adaptive solutions for an aging society.

### 4.2 Key domain-specific technological innovations for China’s “New older adults” market and their key characteristics

Similarly, the adapted method for the classification themes (64) is applied to 31 extracted articles through the PRISMA method for the identification of key domain-specific technological innovations targeting China’s new older adults market and their key characteristics, as indicated in Table 7.

**Table 7:** Classification: domain-specific technological innovations for China’s new older adults market and their key characteristics.

Domain-specific technological innovations for China’s new older adults market and their key characteristics						
RQ2						
References	Domain	Health & wellness technologies	Living environment & safety technologies	Social inclusion & empowerment technologies	Data infrastructure & platform management	Support & operational technologies
Shao et al. (30)	Older adults Education Technology (EdTech)			*Mobile & Digital Learning Platforms*: Systematic learning content; Easy-to-use interface, Intuitive UI, Voice navigation, Features for emotional assurance		
He et al. (36)	Older adults Care Services (ECS)	*Smart Health Monitoring & Wearable Devices:* Real-time health monitoring *Telemedicine & Remote Consultation Systems:* Video consultations	*Smart Home & IoT-Based Care Systems:* Sensors; Monitoring devices		*Service Matching & Digital Marketplaces:* Digital platforms	
Shao et al. (51)	Older adults Care Resource Allocation	*Telemedicine & Remote Consultation Systems:* Chronic disease management				
Yan (37)	Home Older adults Care Industry	*Smart Health Monitoring & Wearable Devices:* Real-time health monitoring; User-friendly design; Integration with platforms & third-party devices	*Smart Home & IoT-Based Care Systems:* Monitoring devices	*Video Communication & Social Connection Systems:* Audio-video monitoring; Mobile app integration; Simplified UI		
Zhou and Ye (38)	Digital Inclusive Finance (FinTech)			*Digital Finance & E-Commerce Integration:* Mobile payment; Low cost; Credibility; Integration with e-commerce and real estate		
Wang et al. (59)	Urban Planning				*GIS & Data-Driven Spatial Planning:* High-resolution spatial data (POI); GIS integration; Predictive site selection	
Sun (41)	Property-based Older adults Care		*AI-Driven Predictive Safety & Resource Allocation:* Predictive modeling; Spatio-temporal data fusion; Dynamic thresholding; Graded early warning			*Privacy-Preserving Computing:* Federated learning
Yingjie and Huayao (42)	Senior Tourism Industry	*Smart Health Monitoring & Wearable Devices:* Real-time health monitoring			*Service Matching & Digital Marketplaces:* Online booking; Feedback mechanisms	*Digital Promotion & Self-Media Platforms:* Use of graphics and short videos for promotion
Yichen (46)	Smart Home Care (SHC) Technology	*Smart Health Monitoring & Wearable Devices:* Real-time health monitoring; User-friendly design; Continuous data collection	*Smart Home & IoT-Based Care Systems:* Sensors, Monitoring devices; Voice recognition; Motion capture; Integrates hardware & software			
Akmal et al. (31)	Older adults Welfare Cottage Industry				*Centralized Data & Information Management Platforms:* Cloud-based centralized databases; Role-based access control	
Jiang (45)	Integrated Medical and Care Service System	*Smart Health Monitoring & Wearable Devices:* Real-time health monitoring *Telemedicine & Remote Consultation Systems:* Rapid telephone consultation; Video consultation; Chronic disease management; Internet medical resources			*Centralized Data & Information Management Platforms:* Health data platform; Four-level linkage mechanism (City, District, Street, Community)	
Tan et al. (32)	Social Service Incentive Mechanisms			*Digital Finance & E-Commerce Integration:* Digital virtual currency/points		
Zheng et al. (39)	Meal Assistance Service	*Intelligent Delivery & Nutrition Management Systems:* Smart meal systems; Online ordering; Nutrition assessment; Delivery dispatch; Multimodal kitchen-terminal tech				
Wan et al. (34)	Agriculture & Digital			*Social Connection & Communication Platforms:* Digital Platforms & Smart Logistics; Social Communication Platforms; Use of Cell Phones, Tablets, and Communication Software *Online Education & Lifelong Learning Platforms:* Platforms for Skills & Education		*Foundational Digital Access Infrastructure:* Basic Connectivity Networks
Chen and Hu (50)	XR Technology			*Extended Reality (XR) & Immersive Technologies:* Gesture Interaction; Optimized Gesture Set for the Older adults	*User-Centered & Aging-Adapted Interaction Design:* High Cognitive Compatibility & Low Physiological Load	
Shen et al. (47)	Healthcare Devices	*Intelligent Health Monitoring & Management Systems:* Smart Wearable Devices	*Assistive Robotics for Daily Living*: Physical Assistance Robots; Social Robots			
Gao et al. (40)	Cultural Technology			*Extended Reality (XR) & Immersive Technologies:* Immersive Learning Environments	*User-Centered & Aging-Adapted Interaction Design:* Adapted Product Presentation (fonts, colors)	*Foundational Digital Access Infrastructure:* Age-Suitable Terminal Products
Zhang et al. (55)	Care Monitoring	*AI-Powered Safety & Behavior Recognition Systems:* Behavior Recognition Technology (using RGB cameras); Deep Learning-based Fall Detection (CNNs, Transfer Learning)				
Lam and Lee (35)	Social Services			*Social Connection & Communication Platforms:* Use of Cell Phones, Tablets, and Communication Software; Social Communication Platforms *Online Education & Lifelong Learning Platforms:* Platforms for Skills & Education		
Cheng et al. (58)	Education			*Social Connection & Communication Platforms:* Social Communication Platforms *Digital Finance & E-Commerce Integration:* Mobile Payment Systems (Alipay, WeChat Pay)		*Foundational Digital Access Infrastructure*: Age-Suitable Terminal Products
Zhang (33)	Tourism			*Online Education & Lifelong Learning Platforms:* E-Learning Platforms for Older Adults	*Data-Driven Analytics & Smart Service Integration:* AI Algorithms for Tourism (Machine Learning, NLP)	
Yu and Dong (48)	Urban Planning				*Data-Driven Analytics & Smart Service Integration:* Geospatial Modeling & Semantic Segmentation; Integrated “micro-clinic + smart pharmacy” networks	
Cheng (57)	Education			*Online Education & Lifelong Learning Platforms:* E-Learning Platforms for Older Adults; Digital Devices for Learning (TVs, mobile phones)		
An et al. (56)	Older adults-Care Services			*Social Connection & Communication Platforms:* Social Communication Platforms *Digital Finance & E-Commerce Integration:* Digital Platforms & Smart Logistics		
Huang et al. (53)	Assistive Robotics		*Assistive Robotics for Daily Living:* Feeding Assistive Robotics (using HCI)			
Hu et al. (49)	Digital Healthcare	*Intelligent Health Monitoring & Management Systems:* Remote Homecare Systems				
Zhang (43)	Education			*Online Education & Lifelong Learning Platforms:* New Media & Multimedia Applications		
Zhao and Gai (60)	Older adults Care Services				*Data-Driven Analytics & Smart Service Integration:* Constrained Clustering Algorithms	
Wang (44)	New Media			*Digital Finance & E-Commerce Integration:* Payment Security Features & Family Account Integration		*Foundational Digital Access Infrastructure:* Simplified Software Interfaces (“Older adults Mode”); Guided Tutorials
Yun (52)	Health & Wellness Technology	*Intelligent Health Monitoring & Management Systems:* Smart Wearable Devices; Multi-Sensor Data Platforms (ECG, pulse oximeter, EMG)			*User-Centered & Aging-Adapted Interaction Design:* User-Centered Design; Intuitive Human-Computer Interaction	
Chen and Chen (54)	Care Technology	*AI-Powered Safety & Behavior Recognition Systems*: Deep Learning-based Fall Detection (CNNs, Transfer Learning)				

#### 4.2.1 Assessment of RQ2: what are the key technological innovations by key industry sectors to serve the “New older adults” market in China, and what are their core characteristics?

The literature, mapped in Table 7, highlights diverse technological innovations tailored to meet the multifaceted needs of China’s new older adults population, also it highlights the key characteristics of these innovations in domains: health and wellness, living environment and safety, social inclusion and empowerment, data infrastructure and platform management, and support and operational technologies.

1. *Health & Wellness Technologies.* Health-related technologies remain the most dominant category. Multiple studies (36, 37, 42, 45–47, 52) emphasize: Smart health monitoring and wearable devices with real-time tracking, multi-sensor platforms (ECG, EMG, pulse oximetry), and older adults-friendly interfaces. Telemedicine and remote consultation systems supporting video consultations, chronic disease management, rapid telephone consultations, and internet medical resources (36, 45, 51). Intelligent health management platforms, including remote homecare systems (49). These innovations prioritize continuous monitoring, accessibility, and early detection, addressing chronic conditions and mobility limitations prevalent among older adults populations.
2. *Living Environment & Safety Technologies.* Studies highlight rapidly advancing smart home safety and environmental monitoring innovations. These include, IoT-based smart home systems with sensors, motion capture, voice recognition, and integrated hardware-software ecosystems (36, 37, 46), AI-driven predictive safety systems, spatio-temporal fusion, dynamic thresholding, and graded early warning models (41), assistive robotics providing physical and social assistance, improving daily living functions (47, 53), and AI-powered fall detection and behavior recognition through deep learning, CNNs, transfer learning, and RGB camera technology (54, 55). This domain reflects a transition from passive to proactive safety ecosystems, improving risk detection and supporting independent living.
3. *Social Inclusion & Empowerment Technologies.* A significant body of literature now focuses on technologies that enhance social connectivity, lifelong learning, and digital participation, like social communication platforms using smartphones, tablets, communication software, and simplified interfaces (35, 37, 56), digital learning and EdTech platforms with intuitive UI, voice navigation, emotion-support features, and accessible e-learning environments (30, 43, 57, 58), XR and immersive learning technologies enabling realistic interactions, gesture-based input, and older adults-optimized cognitive load (40, 50), digital promotion, graphics-led media, and short videos for senior tourism engagement (42).

These technologies support *active aging*, enabling seniors to learn, connect, and participate socially and culturally.

4. *Data Infrastructure & Platform Management.* A strong digital backbone underlies most older adults-oriented innovations, like centralized data and cloud-based information systems (31), multi-level health data platforms linked across city–district–street–community levels (45), digital finance ecosystems, including mobile payments, secure transactions, virtual currency/points, older adults-mode interfaces, and integration with e-commerce and real estate (32, 38, 44), GIS-based urban planning, geospatial modeling, semantic segmentation, and predictive site selection for older adults-friendly development (48, 59), and foundational digital access infrastructure, including older adults-friendly devices and simplified software interfaces (34, 40, 58). This domain shows a movement toward data-driven policymaking, integrated care networks, and inclusive digital finance.
5. *Support & Operational Technologies.* Operational innovations aim to improve service delivery, logistics, and backend efficiency, such as service matching platforms connecting older adults users with care or tourism services (36, 42, 56), intelligent meal assistance and nutrition management systems, including multimodal technologies from ordering to dispatch (39), smart logistics and digital platforms for communication, social support, and product delivery (34, 56), and privacy-preserving computing (federated learning) to ensure secure AI-driven services (41). These technologies enhance *efficiency, personalization, and secure delivery* of community services.

The technological landscape for China’s “new older adults” market is characterized by an integrated ecosystem of health monitoring, smart living environments, tools related to social, educational, and immersive XR, sophisticated data management platforms, and operational support systems. This multi-domain innovations cater to the comprehensive needs of older adults users, balancing health, safety, empowerment, financial inclusion, mobility and quality of life with advanced data-driven management and service delivery models.

### 4.3 Key domain-specific technological innovations for China’s “New older adults” market and their key benefits

Likewise, the adapted method for the classification themes (64) is applied to 31 extracted articles through the PRISMA method for the identification of key domain-specific technological innovations targeting China’s new older adults market and their key benefits, as indicated in Table 8.

**Table 8:** Classification: domain-specific technological innovations for China’s “New older adults” market and their key benefits.

Industry-specific technological innovations for China’s ‘New older adults’ market and their key benefits						
RQ3						
References	Domain	Health & wellness technologies	Living environment & safety technologies	Social inclusion & empowerment technologies	Data infrastructure & platform management	Support & operational technologies
Shao et al. (30)	Older adults Education Technology (EdTech)			*Mobile & Digital Learning Platforms:* Continuous self-education; Integration into society; Social inclusion; Sense of accomplishment		
He et al. (36)	Older adults Care Services (ECS)	*Smart Health Monitoring & Wearable Devices:* Health tracking *Telemedicine & Remote Consultation Systems:* Accessible healthcare; Remote diagnosis	*Smart Home & IoT-Based Care Systems:* Real-time monitoring		*Service Matching & Digital Marketplaces:* Reduces transaction costs	
Shao et al. (51)	Older adults Care Resource Allocation				*Centralized Data & Information Management Platforms:* Improves resource matching	
Yan (37)	Home Older adults Care Industry	*Smart Health Monitoring & Wearable Devices:* Improved safety; Independent living; Real-time alerts; Health tracking	*Smart Home & IoT-Based Care Systems:* Real-time monitoring; Risk prevention; Improved service quality; Remote supervision; Enhances independent living	*Video Communication & Social Connection Systems:* Remote family connection; Emergency detection; Mental support; Easy access to communication	*Centralized Data & Information Management Platforms:* Enables integrated service delivery	
Zhou and Ye (38)	Digital Inclusive Finance (FinTech)			*Digital Finance & E-Commerce Integration:* Accessible & efficient financial services; Easier saving & investing, profitable options		
Wang et al. (59)	Urban Planning				*GIS & Data-Driven Spatial Planning:* Objective site selection for care facilities; Improved spatial equity; Enhanced accessibility	
Sun (41)	Property-based Older adults Care		*AI-Driven Predictive Safety & Resource Allocation:* Early warning of resource shortages; Optimized resource allocation; Supports operational planning			*Privacy-Preserving Computing:* Protects data privacy
Yingjie and Huayao (42)	Senior Tourism Industry				*Centralized Data & Information Management Platforms:* Improves resource matching; Enables integrated service delivery *Service Matching & Digital Marketplaces:* Facilitates comparison & selection of services	*Digital Promotion & Self-Media Platforms:* Increases public awareness; Promotes culture & services; Boosts engagement
Yichen (46)	Smart Home Care (SHC) Technology	*Smart Health Monitoring & Wearable Devices:* Improved safety; Independent living; Reduced hospitalization	*Smart Home & IoT-Based Care Systems:* Real-time monitoring; Risk prevention; Improved service quality; Remote supervision; Enhances independent living			
Akmal et al. (31)	Older adults Welfare Cottage Industry	*Telemedicine & Remote Consultation Systems:* Accommodates rural service imbalances			*Centralized Data & Information Management Platforms:* Better care coordination; Accessible anywhere	
Jiang (45)	Integrated Medical and Care Service System	*Smart Health Monitoring & Wearable Devices:* Real-time alerts; Health tracking *Telemedicine & Remote Consultation Systems:* Accessible healthcare; Remote diagnosis; Reduces travel & cost			*Centralized Data & Information Management Platforms:* Improves resource matching; Enables integrated service delivery	
Tan et al. (32)	Social Service Incentive Mechanisms				*Service Matching & Digital Marketplaces:* Improves purchasing ability; Facilitates comparison & selection of services	
Zheng et al. (39)	Meal Assistance Service	*Intelligent Delivery & Nutrition Management Systems:* Enhanced convenience; Better dietary matching; Improved nutrition; Supports sustainable operation				
Wan et al. (34)	Agriculture & Digital			*Social Connection & Communication Platforms:* Brings convenience; Reduces depression by narrowing the urban–rural living standards gap; provides “remote companionship”; Reduces loneliness; Improves cognitive ability; Offers access to mental health knowledge *Online Education & Lifelong Learning Platforms:* Increases income; Enhances sense of career accomplishment		*Foundational Digital Access Infrastructure:* Provides the foundational access that enables all other digital services; Reduces the “digital divide”
Chen and Hu (50)	XR Technology			*Extended Reality (XR) & Immersive Technologies:* Intuitive and low learning curve; Enhanced quality of life; Promotes independence and social connection; Reduced learning difficulty; Reduced risk of musculoskeletal disorders	*User-Centered & Aging-Adapted Interaction Design:* Makes the technology easier to adopt; Minimizes physical strain from repetitive use	
Shen et al. (47)	Healthcare Devices	*Intelligent Health Monitoring & Management Systems:* Dynamic health information; Improved quality of life, physical, and mental health; Immediate assistance (e.g., fall detection)	*Assistive Robotics for Daily Living:* Assist with activities of daily living; Improve gait function; Promote independence and convenience; Maintain social connections; improve spiritual life			
Gao et al. (40)	Cultural Technology			*Extended Reality (XR) & Immersive Technologies:* Helps deepen the learning and application of digital cultural products	*User-Centered & Aging-Adapted Interaction Design:* Makes products more suitable for the older adults	*Foundational Digital Access Infrastructure:* Improved convenience for the older adults to enter the online world
Zhang et al. (55)	Care Monitoring	*AI-Powered Safety & Behavior Recognition Systems:* High accuracy and speed in recognition; Improved service quality in older adults care; Timely detection of abnormal situations (e.g., falls); Enhanced safety and security in everyday life				
Lam and Lee (35)	Social Services			*Social Connection & Communication Platforms:* Improves cognitive ability; Provides “remote companionship”; Reduces loneliness. *Online Education & Lifelong Learning Platforms:* Enhances sense of career accomplishment		
Cheng et al. (58)	Education			*Social Connection & Communication Platforms:* Provides “remote companionship”; Reduces loneliness; Maintains social connections *E-commerce & Mobile Payment Ecosystems:* Mobile Payment Systems (Alipay, WeChat Pay); Provides convenience		*Foundational Digital Access Infrastructure:* Improved convenience for the older adults to enter the online world
Zhang (33)	Tourism			*Online Education & Lifelong Learning Platforms:* Maintained lifelong learning from home	*Data-Driven Analytics & Smart Service Integration:* Precise marketing and personalized travel packages	
Yu and Dong (48)	Urban Planning				*Data-Driven Analytics & Smart Service Integration:* Analyzes spatial characteristics; Predicts optimal sites for older adults care facilities; Assesses walkability; Aims to enhance basic healthcare provision in underserved rural areas	
Cheng (57)	Education			*Online Education & Lifelong Learning Platforms:* Provided access to high-quality educational resources; Maintained lifelong learning from home; Enabled flexible; Enabled self-arranged learning time and content		
An et al. (56)	Older adults-Care Services			*Social Connection & Communication Platforms:* Maintains social connections *E-commerce & Mobile Payment Ecosystems:* Brings convenience		
Huang et al. (53)	Assistive Robotics		*Assistive Robotics for Daily Living:* Promotes independence			
Hu et al. (49)	Digital Healthcare	*Intelligent Health Monitoring & Management Systems:* Allows ageing in place; Enables professional care at home; Reduces hospitalizations and readmission rates through early detection; Enables self-monitoring and management of chronic diseases				
Zhang (43)	Education			*Online Education & Lifelong Learning Platforms:* Creates new opportunities for the sustainable development of older adults education; Helps the older adults learn new knowledge, master new technologies, and integrate into society		
Zhao and Gai (60)	Older adults Care Services				*Data-Driven Analytics & Smart Service Integration:* Improved efficiency & quality for industrial chain optimization; Optimal resource allocation to efficiently connect caregivers with the older adults	
Wang (44)	New Media			*E-commerce & Mobile Payment Ecosystems:* Reduced property security risk; Enabling online shopping despite a lack of digital payment confidence		*Foundational Digital Access Infrastructure:* Enhanced usability and reduced complexity; Improved learnability and confidence
Yun (52)	Health & Wellness Technology	*Intelligent Health Monitoring & Management Systems:* Dynamic health information Improved quality of life, physical, and mental health; Comprehensive health monitoring			*User-Centered & Aging-Adapted Interaction Design:* Accommodates the older adults’s physical and psychological state; Natural, easy-to-use, and humanized product interfaces	
Chen and Chen (54)	Care Technology	*AI-Powered Safety & Behavior Recognition Systems:* Timely detection of abnormal situations (e.g., falls); Enhanced safety and security in everyday life; Aims to ensure the safety of the older adults living alone				

#### 4.3.1 Assessment of RQ3: what are the key benefits of these technological innovations for China’s “New older adults” population?

The literature, mapped in Table 8, highlights how technological innovations across various industries provide following tailored benefits to China’s new older adults market.

1. *Health & Wellness Technologies*. A wide range of studies emphasize how smart health monitoring, wearable devices, and telemedicine strengthen older adults health management. Smart health systems (36, 37, 45–47, 49, 52) provide real-time tracking, early alerts, fall detection, and chronic disease management, collectively reducing hospitalization and enabling ageing in place. Telemedicine and remote consultation systems (31, 36, 45) expand access to healthcare by reducing travel costs and supporting remote diagnosis, particularly beneficial for rural older adults facing service shortages.
2. *Living Environment & Safety Technologies.* Smart home, IoT-based systems, AI-driven predictive safety tools, and assistive robotics significantly enhance older adults safety and independence. Yan (37), He et al. (36), Yichen (46), Sun (41), and Shen et al. (47) highlight benefits such as real-time monitoring, risk prevention, remote supervision, and early warning of hazards or resource shortages. Care robotics (47, 53) further assist with activities of daily living, improving gait, convenience, and autonomy. These technologies create safe, supportive living environments while improving service quality.
3. *Social Inclusion & Empowerment Technologies.* Digital platforms supporting lifelong learning, social connection, and communication play a vital role in reducing loneliness, enhancing mental well-being, and empowering the older adults. Studies such as Shao et al. (30), Yan (37), Lam and Lee (35), and Cheng (57, 58) show that mobile learning platforms and online education expand access to high-quality content, support digital literacy, and foster a sense of accomplishment. Social communication systems (34, 35, 42) provide remote companionship, reduce depression, and maintain familial or social ties. Meanwhile, digital marketplaces (32, 42) enhance empowerment by enabling informed service comparison and better purchasing decisions. Extended Reality (XR) technologies (40, 50) further promote social and cultural participation by offering intuitive, immersive experiences suited to older adults users.
4. *Data Infrastructure & Platform Management.* Centralized, cloud-based, and data-driven platforms facilitate resource integration, care coordination, and efficient service delivery. Shao et al. (51), Yan (37), Jiang (45), Akmal et al. (31), and Yingjie and Huayao (42) show that shared health and service data platforms ensure smoother multi-agency collaboration and accurate resource allocation. GIS-based planning tools (48, 59) optimize the placement of care facilities, improving accessibility and spatial equity. Digital finance and e-commerce ecosystems (38, 44, 56) provide secure, efficient financial services, fostering economic inclusion for older adults users who may lack digital payment confidence.
5. *Support & Operational Technologies*. Supportive and operational technologies improve service efficiency, convenience, and trust. Intelligent delivery and nutrition management systems (39) ensure accurate dietary matching and support sustainable meal services. Privacy-preserving computing (41) protects sensitive personal data in digitized service environments. Service matching platforms (32, 36, 42) reduce transaction friction, streamline elder-caregiver matching, and lower operational costs. AI-powered behavior recognition systems (54, 55) enhance security by detecting risky situations such as falls or abnormal movements in real time.

To conclude, domain-specific technological innovations for China’s new older adults market deliver multifaceted benefits that enhance health outcomes, safety, social inclusion, lifelong learning, reduce loneliness, improve social participation, resource integration, and operational efficiency. These innovations are instrumental in supporting independent living, improving care quality, enabling financial and social empowerment, and optimizing service delivery in a rapidly aging society.

### 4.4 Limitations and quality assessment score

It is essential for a PRISMA-compliant Systematic Literature Review (SLR) to evaluate the limitations of studies selected for the review and assess their quality in order to ensure the validity of the findings of the review, transparency in the review process, reducing the risk of bias, and clarifying the scope of the conclusion. Table 9 demonstrates the limitations and quality assessment scores of each of the 31 selected studies.

**Table 9:** Limitations and quality assessment score.

Classification					Quality assessment				
References	Publication channel	Journal/ conference ranking	Cited by	Limitations	a	b	c	d	Scores
Shao et al. (30)	Conference	0	0	Methodologically, the study is limited by its single-province sample and reliance on self-reported survey data, as a result, its findings have restricted generalizability, particularly regarding mobile learning in education.	1	1	−1	0	1
He et al. (36)	Journal	Q1	1	Missing data from certain provinces constrain the regional representation and robustness of the study, and this limits its generalizability and prevents analysis of long-term effects, data privacy, and cross-sector consumption behaviors.	1	1	0	2	4
Shao et al. (51)	Conference	0	0	By focusing only on Dezhou and online respondents, the study’s methodological scope is narrow, as a result, findings cannot be generalized to broader regional older adults-care preferences.	1	1	−1	0	1
Yan (37)	Thesis	0	2	The early-stage exploration lacks mature theoretical frameworks and empirical validation, and its conclusions are limited to pilot projects in two cities and do not reflect broader market segmentation or autonomous older adults behavior.	1	1	0	0	2
Zhou and Ye (38)	Journal	Q1	12	RDD estimates are valid only near the cutoff, limiting methodological generalizability, and this constrains insights into broader market segmentation, non-FinTech innovations, and cross-sector older adults consumption.	1	1	1	2	5
Wang et al. (59)	Journal	Q1	11	The model does not differentiate older adults-care facility types, reducing predictive precision, as a result, the study offers limited insights beyond GIS-based spatial planning.	1	0.5	1	2	4.5
Sun (41)	Journal	Q1	0	Exclusion of rural property data and resident preferences limits the study’s methodological completeness, and this restricts generalizability and the applicability of findings to broader pilot counties or extreme conditions.	1	1	−1	2	3
Yingjie and Huayao (42)	Thesis	0	1	Lack of field validation and empirical support reduces the reliability of conclusions about TCM–tourism integration, and findings are limited by the study’s exclusive macro-level, supply-side focus, omitting older adults behavioral data.	1	0.5	0	0	1.5
Yichen (46)	Dissertation	0	0	The Shanghai-specific sample and short observation window limit methodological robustness, as a result, the findings have constrained generalizability and practical relevance.	1	1	−1	0	1
Akmal et al. (31)	Journal	0	0	Conceptually, the proposed system lacks empirical grounding and cost or technology assessments because it provides no insights into market segmentation, industry practices, or older adults consumption behaviors.	1	1	−1	0	1
Jiang (45)	Journal	0	0	The qualitative, location-specific approach limits methodological applicability to other regions, as a result, policy and institutional findings cannot be generalized to the broader older adults market or technology landscape.	1	1	−1	0	1
Tan et al. (32)	Journal	0	0	Purposive sampling and self-reported measures constrain methodological representativeness, and it focuses narrowly on a points-based system for low-income individuals, ignoring broader sectoral or market insights.	1	1	−1	0	1
Zheng et al. (39)	Journal	Q1	0	Omitting key variables reduces the methodological completeness of the model, which limits exploration of consumer technology adaptation or detailed behavioral analysis.	1	0.5	−1	2	2.5
Wan et al. (34)	Journal	Q1	2	Lack of theoretical grounding and empirical testing restricts methodological robustness, as a result, rural micro-level findings cannot be generalized nationally, and causation cannot be inferred.	1	1	0	2	4
Chen and Hu (50)	Journal	Q2	0	Data-based evaluation without theoretical grounding weakens methodological support, and its XR gesture focus limits insights into other industries, market segmentation, or older adults behaviors.	1	1	−1	1.5	2.5
Shen et al. (47)	Journal	Q1	18	Limited indicators and lack of geographic validation reduce methodological robustness, and findings from two nursing homes cannot generalize to diverse older adults populations or sectors.	1	1	1	2	5
Gao et al. (40)	Conference	0	3	Narrow sample scope and insufficient field validation limit methodological reliability, and its focus on digital cultural products lacks cross-sector analysis.	1	1	0	0	2
Zhang et al. (55)	Journal	Q3	0	Sparse older adults-behavior data and limited GIS variables reduce methodological accuracy, as a result, findings focus only on technical monitoring and not broader market or behavioral insights.	1	1	−1	1	2
Lam and Lee (35)	Conference	Q3	9	Absence of longitudinal data constrains methodological reflection of dynamic needs, and convenience sampling further limits generalizability, focusing only on intention rather than actual use.	1	1	1	1	4
Cheng et al. (58)	Journal	Q2	34	Insufficient differentiation between facility types weakens methodological precision, and its single-case focus is not generalizable to industry strategies or older adults market behavior.	1	1	1	1.5	4.5
Zhang (33)	Journal	Q3	11	Limited data sources and lack of multi-city validation reduce methodological reliability, and its tourism-sector focus prevents cross-sector or regional insights.	1	1	1	1	4
Yu and Dong (48)	Journal	Q2	1	Reliance on selected factors limits predictive completeness, and using GIS and POI data, the study omits socioeconomic and user-experience variables.	1	0.5	0	1.5	3
Cheng (57)	Journal	Q1	0	Simulation-based methods with unverified assumptions restrict methodological validity, and small rural samples and education-only focus prevent generalization to broader older adults markets.	1	0.5	−1	2	2.5
An et al. (56)	Journal	Q1	0	Incomplete service-quality indicators reduce methodological comprehensiveness, and findings are limited to one community intervention, with no sectoral or commercial relevance.	1	1	−1	2	3
Huang et al. (53)	Conference	0	3	Lack of empirical testing across cities limits methodological generalizability, and focusing on a single robotic device provides no market, adoption, or user behavior insights.	1	1	0	0	2
Hu et al. (49)	Journal	Q4	3	Limited urban samples and insufficient model validation constrain methodological robustness, and findings from digital health in nursing homes cannot be extrapolated to other sectors or older adults behaviors.	1	1	0	1	3
Zhang (43)	Conference	0	0	Preliminary indicator framework and limited expert validation weaken methodological reliability, and its education-sector focus excludes cross-sector insights into segmentation or technology adaptation.	1	1	-1	0	1
Zhao and Gai (60)	Journal	Q3	2	Use of selected spatial variables oversimplifies methodological complexity, and supply-side, industry-chain focus prevents understanding of older adults behavior or cross-sector differences.	1	1	0	1	3
Wang (44)	Conference	0	0	Failure to incorporate behavioral, social, and psychological factors limits methodological explanatory depth, and urban self-reported sample restricts representativeness and sectoral applicability.	1	1	-1	0	1
Yun (52)	Conference	0	0	Limited geographic scope and lack of cross-regional comparison reduce methodological applicability, and conceptual design without user data prevents insights into market segmentation or broader health technology use.	1	1	-1	0	1
Chen and Chen (54)	Conference	0	0	Omitting dynamic service-allocation factors restricts methodological guidance for policy, and narrow engineering focus on a single fall-detection algorithm provides no broader market, sectoral, or behavioral insights.	1	1	-1	0	1

#### 4.4.1 Potential limitations

This review is limited by the methodological constraints of the included studies. Many studies focus on specific cities, provinces, or pilot sites, relying on small, localized samples, qualitative case studies, or online surveys, often excluding non-digital users. Additionally, a significant portion of the evidence is derived from theoretical frameworks, macro-level analyses, or models with limited empirical validation. These methodological limitations restrict the generalizability of findings and prevent the review from fully addressing broader market segmentation, cross-sector consumption patterns, long-term effects, and practical implementation challenges in older adults-care research.

#### 4.4.2 Overall quality assessment score

According to Farooq et al. (28), the minimum possible score for the study is −1, and the maximum possible score is 5. The average score is calculated as approximately 2.4 in the case of this review. The results of quality assessment in Table 10, indicate that most included studies, i.e., 52% of the included studies, scored above the average quality score of 2.4, which indicates a reasonable proportion of methodologically robust research. In contrast, 48% fell below this threshold, highlighting substantial limitations, which are common in developing fields. Overall, these results suggest that the review includes several high-quality studies. This range of scores offers a realistic and comprehensive view, allowing for conclusions that are strengthened by synthesizing diverse perspectives while rightly prioritizing the most rigorous contributions.

**Table 10:** Overall quality assessment score.

References	Score	Total	Percentage	
Shen et al. (47) and Zhou and Ye (38)	5	2	6%	52%
Cheng et al. (58) and Wang et al. (59)	4.5	2	6%	
He et al. (36), Lam and Lee (35), Wan et al. (34) and Zhang (33)	4	4	13%	
An et al. (56), Hu et al. (49), Sun (41), Yu and Dong (48) and Zhao and Gai (60)	3	5	16%	
Chen and Hu (50), Cheng (57) and Zheng et al. (39)	2.5	3	10%	
Gao et al. (40), Huang et al. (53), Yan (37) and Zhang et al. (55)	2	4	13%	48%
Yingjie and Huayao (42)	1.5	1	3%	
Akmal et al. (31), Chen and Chen (54), Jiang (45), Shao et al. (30), Shao et al. (51), Tan et al. (32), Wang (44), Yichen (46), Yun (52) and Zhang (43)	1	10	32%	

## 5 Discussion

The findings from this systematic review illustrate the complexity and heterogeneity of China’s new older adults market, emphasizing the importance of domain-specific segmentation to effectively engage this rapidly growing demographic. The reviewed studies demonstrate that older adults consumers differ across multiple dimensions, including demographics, geography, psychographics, health status, and economic capacity. This multidimensional segmentation reflects the diversity of needs, preferences, and behaviors among older adults, enabling more tailored offerings such as digital education platforms for lifelong learning, IoT-based home care for independent living, and accessible financial technologies. Recognizing this heterogeneity underscores the necessity of flexible, multi-faceted strategies, rather than “one-size-fits-all” approaches, to address differences in lifestyle, health conditions, digital literacy, and generational attitudes.

Technological innovations targeting the new older adults market are both sophisticated and multifaceted, spanning health and wellness, living environment and safety, social inclusion, data infrastructure, and operational support. Examples include wearable health devices, telemedicine platforms, smart home systems, XR-based learning environments, and integrated digital finance and care platforms. These innovations prioritize accessibility and ease of use, accommodating varying levels of digital literacy, while advanced applications of AI, big data, and cloud computing enable predictive care, personalized service delivery, and efficient resource allocation. Collectively, these technologies not only enhance quality of life, independence, and social participation among older adults but also contribute to broader societal objectives such as reducing healthcare system burdens, promoting digital inclusion, and fostering lifelong learning and empowerment.

Despite these promising developments and including a reasonable proportion of methodologically robust research, significant challenges remain. The geographic and demographic scope of much existing research is limited, with a predominant focus on urban, digitally literate populations, often overlooking rural, low-income, or digitally marginalized groups. Additionally, many studies rely on theoretical frameworks, macro-level analyses, or small-scale empirical studies, constraining understanding of actual adoption behaviors, long-term outcomes, and real-world impact. Addressing these gaps requires more inclusive, empirical, and longitudinal research to inform scalable, equitable, and context-sensitive interventions capable of meeting the complex and evolving needs of China’s aging population.

Overall, this review highlights that China’s new older adults market is not a homogeneous group but a multidimensional population whose health, social, economic, and technological needs are diverse. Effective strategies must integrate domain-specific segmentation, technological innovation, and inclusive service design to support independent living, enhance well-being, and enable active participation in an increasingly digital society.

## 6 Conclusion

This review demonstrates that China’s new older adults market is highly heterogeneous, with diverse needs across demographics, geography, psychographics, health, and economic capacity, and an effective engagement requires domain-specific, technology-enabled solutions, like wearable health devices, smart home systems, telemedicine platforms, and digital learning tools in order to prioritize accessibility, usability, and personalization. The findings highlight the importance of multidimensional segmentation and user-centered design to support independent living, enhance health and safety, foster social participation, and improve overall quality of life, along with tailored interventions that account for generational differences, urban–rural disparities, digital literacy, health status, and lifestyle preferences are essential for developing inclusive, efficient, and sustainable care and engagement systems for older adults.

### 6.1 Limitations and future recommendations

#### 6.1.1 Limitations

This review is subject to several notable limitations, such as the geographic focus of many studies is predominantly urban or region-specific, limiting the generalizability of findings to rural, less-developed, or ethnically diverse areas. Also, a substantial proportion of the literature centers on digitally literate or tech-savvy older adults populations, often excluding non-digital users and thereby potentially skewing insights toward more connected subsets of the older adults, and many studies rely on theoretical frameworks, macro-level analyses, or small-scale case studies, providing limited empirical evidence of actual adoption behaviors, preferences, and long-term outcomes. All of these methodological constraints restrict understanding of cross-sector consumption patterns, real-world effectiveness, and practical implementation challenges, and lastly the heterogeneity of China’s new older adults population, in terms of health status, lifestyle, generational differences, and economic capacity, remains underexplored in many studies, highlighting the need for more inclusive, longitudinal, and context-sensitive research to inform scalable and equitable interventions.

#### 6.1.2 Future recommendations

Future efforts should focus on actionable strategies aligned with market segmentation and technological innovation, and businesses and service providers should develop differentiated offerings targeting subgroups with distinct motivations, such as self-improvement versus comfort-focused older adults, while considering urban–rural and income-based differences. On the other hand, technology developers should prioritize user-friendly designs, including simplified interfaces, voice navigation, and adaptive settings, ensuring accessibility for all older adults users. In the same way, policymakers should incentivize inclusive solutions, promote digital literacy, and support integration across health, social, financial, and educational domains. In the end, longitudinal and empirical research, particularly among rural and digitally marginalized populations, is needed to evaluate real-world adoption, long-term outcomes, and cross-sector integration, while co-design approaches involving older adults users can ensure innovations are aligned with actual needs and preferences.

## Data Availability

The original contributions presented in the study are included in the article/supplementary material, further inquiries can be directed to the corresponding author.
